# Effect of Phase Composition on Leaching Behavior and Mechanical Properties of Ceramics from Ferrochrome Slag and Tundish Slag

**DOI:** 10.3390/ma15061993

**Published:** 2022-03-08

**Authors:** Dejian Pei, Yu Li, Xiangjie Duan, Daqiang Cang, Yindong Yang, Alex McLean, Zhancheng Guo, Chuanhua Xu

**Affiliations:** 1 State Key Laboratory of Advanced Metallurgy, University of Science and Technology Beijing, Beijing 100083, China; peidejian1990@126.com (D.P.); duanxiangjie@gmail.com (X.D.); cangdaqiang@metall.ustb.edu.cn (D.C.); zcguo@ustb.edu.cn (Z.G.); 2 Sinosteel Maanshan General Institute of Mining Research Co., Ltd., Maanshan 243000, China; xuch2@sinosteel.com; 3 Department of Materials Science and Engineering, University of Toronto, 184 College Street, Toronto, ON M5S 3E4, Canada; yy9861@yahoo.com (Y.Y.); amclean16@cogeco.ca (A.M.)

**Keywords:** pyroxene/spinel, ceramics, Cr/Mn leaching, mechanical properties, cleaner industrial chain

## Abstract

Ferrochrome slag (FS) and tundish slag (TS) are two typical slags containing high contents of Cr_2_O_3_ (3.88 wt.%) and MnO (18.69 wt.%), respectively. In this study, batches of ceramics were prepared from FS and TS, and their Cr/Mn leaching behaviors, mechanical properties and microstructures were investigated. Results showed that ceramics with 80 wt.% FS or 85 wt.% TS had acceptable properties. By controlling its composition and sintering temperature, pyroxene or spinel phases could become the main crystalline phases of the fired ceramics containing either of the two slags. For both slag series, pyroxene phases contributed to higher bending strengths, whereas spinel phases led to lower Cr/Mn leaching rates. Both ceramic containing 20 wt.% FS and ceramic containing 85 wt.% TS had the main crystals of pyroxene phases and possessed the highest bending strengths (FS20: 114.52 MPa and TS85: 124.61 MPa). However, both ceramic containing 80 wt.% FS and ceramic containing 25 wt.% TS with main crystals from the spinel phases had the lowest Cr/Mn leaching rates (FS80: Cr 0.05% and TS25: Mn 0.43%). Therefore, optimum designs for the compositions of ceramics from different slags were achieved by changing the proportions of pyroxene and spinel phases to obtain a balance between the high strengths of materials and the stable retention of heavy metal ions. This study provides an important basis for long-term research on the large-scale reuse of heavy metal-containing slags in the ceramic industry.

## 1. Introduction

The clean and economical disposal of slags containing heavy metals is essential for an eco-friendly society. Ferrochrome slag (FS) and tundish slag (TS) are two typical slags with high contents of heavy metals. FS is a bulky by-product of the ferrochromium industry. Global FS production was approximately 11.7 million tons in 2017, and it has also been expected to increase by about 3.6% annually [1]. Recycling and the efficient usage of large amounts of FS are significant challenges for ferrochrome producers because of the high content of Cr_2_O_3_ in FS. Research on the utilization of FS has been broadly classified under the following categories: (1) refractory applications, such as refractory castables [2]; (2) applications in the construction of roads and buildings [3] and as aggregates in concrete [4]; and (3) applications in the treatment of hazardous hexavalent Cr [5], for example, in the removal of hexavalent Cr from jigging water streams. TS is discharged from tundish after the continuous casting of steel. Few studies have been reported on the reuse of TS. According to an investigation performed at Shandong Steel in China, approximately 2000 tons of TS are released from a workshop every year and accumulate in landfills without being reused. A high content of Mn (18.69 wt.%) was detected in TS in 2015, and this result is similar to that reported in the literature [6]. The ceramic industry is the third-largest silicate industry after the cement and concrete industries, and these bulk raw materials can be utilized in the ceramic industry to improve their added value. Ceramics have an excellent solidification ability in relation to heavy metals compared with cement or concrete, and building ceramics, especially exterior wall tiles and outdoors ground tiles, are consumed in a large quantity and have a lower environmental impact than those other indoors materials, and they also have the potential to be the main product for the utilization of those metallurgic slags. However, research on the reuse of FS and TS as raw materials in the ceramic industry has rarely been reported.

Recently, Zhao and Jiang et al. [7,8,9,10] synthesized ceramics with high contents of CaO and Fe_2_O_3_ from solid waste, including steel slag, tailings and red mud. The main crystals of these ceramics were pyroxene group minerals instead of quartz or mullite. Pyroxene ceramics exhibit mechanical properties with a bending strength of 107–143 MPa, which is significantly higher than those of the raw materials of porcelain (ISO 13006: 2012: ≥35 MPa). Moreover, Na^+^ can be retained in pyroxene and anorthite ceramics prepared from red mud, as these ceramics have low Na^+^ leaching rates [11]. However, only a few studies have been conducted on the Cr/Mn leaching behaviors of pyroxene ceramics. Cr and Mn are toxic elements that affect neurological and muscle functions in humans. Some researchers have proposed that Cr is the most stable in the spinel phase in stainless steel slag because of its strong bonding in the spinel structure, particularly in MgCr_2_O_4_, and the elimination of Cr from stainless steel slag is hindered by the presence of Cr in the spinel structure [12,13]. The ionic radii of Mn^2+^, Fe^2+^, and Zn^2+^ were different in the spinel structure (Mn_0.66_Zn_0.25_Fe_2.09_O_4_), which is highly resistant to acid dissolution [14]. Thus, the fabrication of pyroxene/spinel ceramics can provide a feasible method for obtaining ceramics with suitable mechanical properties and prevent Cr/Mn leaching. 

Based on the above, the specific objectives of this work are to (a) demonstrate the feasibility of producing building ceramics from these metallurgical slags by evaluating their Cr/Mn leaching behaviors and mechanical properties, (b) explore the relationships between the properties and phase/microstructures of the ceramics; and (c) provide some preliminary data for long-term research on the reuse of heavy metal-containing slags in the ceramic industry. X-ray fluorescence (XRF) was used to evaluate the chemical compositions of raw materials and phase and microstructure evolutions were investigated using X-ray diffraction (XRD), scanning electron microscopy (SEM) and energy dispersive X-ray spectroscopy (EDS). 

## 2. Experimental

### 2.1. Raw Materials and Analysis

**Figure 1:**
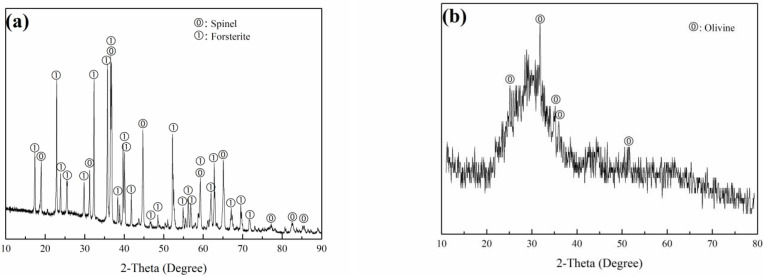
XRD patterns of (**a**) ferrochrome slag and (**b**) tundish slag.

FS and TS were obtained from Qinghai Shanchuan Ferro-alloy Group Co., Ltd. (Xining, China) and Shandong Iron and Steel Group Co., Ltd. (Jinan, China), respectively. Other raw materials were clay, feldspar and quartz, which were acquired from Shandong Province, and dolomite was obtained from Guizhou Province. Chemical compositions and XRD patterns of FS and TS are presented in Table 1 and Figure 1, respectively. The primary phase components of FS are forsterite Mg_2_SiO_4_ and spinel Mg (Al, Cr)_2_O_4_, and those of TS are olivine (Mg_0.028_Fe_0.908_Mn_0.064_)(Mg_0.028_Fe_0.892_Mn_0.057_Ca_0.023_) SiO_4_ and amorphous phases.

Chemical compositions of the raw materials were determined by employing XRF analysis (Shimadzu, XRF-1800, Kyoto, Japan). XRD patterns of the samples were acquired using a Mac M21X powder diffractometer (Rigaku Corporation, Tokyo, Japan). Micromorphological characteristics of the samples were examined using SEM at 25 kV (Carl Zeiss, EVO18 Special Edition, Oberkochen, Germany). The element contents of the leaching solution were measured using an OPTIMA 7000DV ICP-OES (PerkinElmer, Waltham, MA, USA).

### 2.2. Formula Design

Pyroxene/spinel ceramics were prepared by designing the compositions in the crystallization regions of the pyroxene/spinel phases of SiO_2_-Al_2_O_3_-CaO-MgO (MnO) [15]. The presence of other minor components was ignored. The proportions of the raw materials are listed in Table 2. FS20 and TS85 compositions belonged to the crystallization regions of the pyroxene phases, whereas FS80 and TS25 compositions corresponded to the crystallization regions of the spinel phases. The added proportions of FS and TS were changed to design other compositions belonging to the transitional crystallization region between the crystallization regions of the pyroxene and spinel phases. Because of the low plasticity of FS and TS, FS100 and TS100 were not considered in the formula.

### 2.3. Experimental Procedure

Batches (500 g each) shown in Table 2 were mixed and wet-milled (with 500 mL water) in a pot mill for 20 min to achieve homogenization and appropriate fineness. The obtained slurry was oven-dried at 110 °C for 10 h. Then, the agglomerate was broken into fine particles that can pass through a 0.180 mm sieve with a moisture content of 6–8 wt.%. Samples with dimensions of 50 mm × 10 mm × 5 mm were hydraulically compacted using uniaxial pressing under 30 MPa. The shaped samples were dried at 110 °C for 2 h. The dried samples were fired at different sintering temperatures (800–1290 °C) in an electrically operated laboratory furnace at a constant heating rate of 5 °C/min, maintained at the sintering temperature for 30 min, and then cooled to room temperature (28–31 °C) in the furnace.

### 2.4. Physical Properties and Element Leaching Rates

Physical properties of the fired samples were evaluated by conducting water absorption and bending strength (three-point bending) tests in accordance with (ISO 13006: 2012.) Leaching was performed in accordance with the Chinese National Standard (HJ 557–2010), and the procedure was similar to the US EPA SW 846 Method 1311 toxicity characteristics leaching procedure. The specific leaching conditions and steps were as follows: a) A sample with a dry basis weight of 100 g, was placed in a 2 L extraction bottle, and the calculated liquid–solid ratio was 10:1 (L/kg) according to the moisture content of the sample. After adding the extractant into the bottle, closing the bottle cap and fixing vertically on the horizontal shaking device, the shaking frequency was adjusted to 110 times/min and the amplitude to 40 mm. After shaking at room temperature for 8 h, the extraction bottle was removed and kept for 16 h. b) A 0.45 μm microporous filter membrane was installed on the pressure filter, the mixture filtered, the leaching liquid collected and stored according to the requirements of the analysis methods of each analyte.

Chinese regulatory limits for the integrated wastewater discharge standard (GB 8978-1996) were used to assess the leaching toxicity results. The element leaching rate was used to determine the stability of an element and was equal to the ratio of the element content in the leaching solution to that in the sample.

## 3. Results and Discussion

### 3.1. Typical Samples

#### 3.1.1. Crystal Evolution during Sintering Process

**Figure 2:**
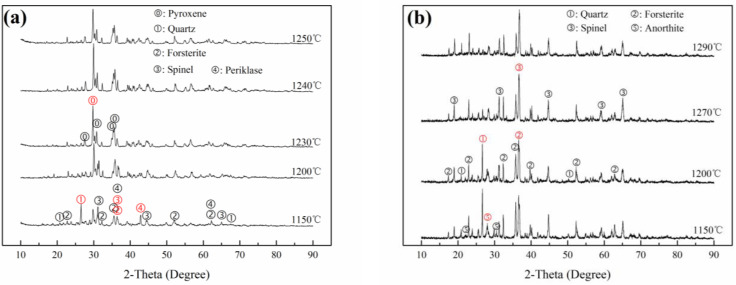
XRD patterns of (**a**) FS20 and (**b**) FS80 sintered at different temperatures.

XRD patterns of FS20, FS80, TS85 and TS25, sintered at different temperatures, are shown in Figure 2.

**Figure 3:**
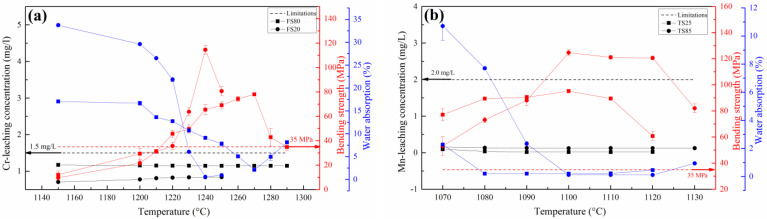
Bending strengths, water absorptions and Cr/Mn leaching concentrations of (**a**) FS20 and FS80 and (**b**) TS25 and TS85 sintered at different temperatures.

**Figure 4:**
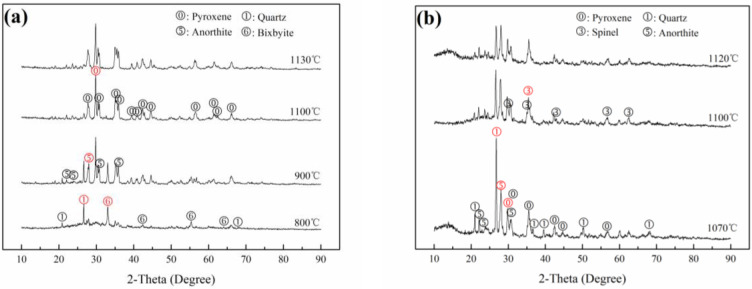
XRD patterns of (**a**) TS85 and (**b**) TS25 sintered at different temperatures.

Pyroxenes, including diopside CaMg(SiO_3_)_2_ and augite (Mg,Fe,Al,Cr)(Ca,Fe,Mn,Mg) (Si,Al)_2_O_6_, were the main crystalline phases in FS20 sintered at 1240 °C (Figure 2). Spinel Mg (Al,Cr)_2_O_4_ was the primary crystalline phase in FS80 sintered at 1270 °C, arising from the original phase in FS and the phase generated during sintering. With an increase in the sintering temperature, the amounts of other phases, such as quartz SiO_2_, forsterite Mg_2_SiO_4_, anorthite CaAl_2_Si_2_O_8_ and periclase MgO, slightly decreased, or these phases disappeared. Note that although quartz transformed at 1290 °C (FS80), its properties did not significantly change (Figure 3 and Figure 4). Similarly, pyroxenes (including diopside manganoan and augite) were the main crystalline phases in TS85 sintered at 1100 °C, and spinel MnAl_2_O_4_-spinel; jacobsite (Mn_0.6_Fe_0.4_) (Mn_0.4_Fe_1.6_)]O_4_ and anorthite were the primary crystalline phases in TS25 sintered at 1100 °C (Figure 4). With an increase in the sintering temperature (≥900 °C), the amounts of other phases such as amorphous phase, bixbyite FeMnO_3_, quartz SiO_2_, anorthite CaAl_2_Si_2_O_8_, and pyroxene [(diopside aluminum; augite) slightly decreased, or these phases disappeared in TS25.

Therefore, it was confirmed that pyroxene and spinel were produced and became one of the main crystalline phases in ceramics (FS20, FS80, TS25 and TS85) when the compositions of ceramics were designed in the crystallization regions of the pyroxene/spinel phases and the ceramics were produced via sintering.

#### 3.1.2. Physical Properties and Cr/Mn Leaching Concentrations

Variations in the water absorptions, bending strengths and Cr/Mn leaching concentrations of FS20, FS80, TS25 and TS85 with respect to the sintering temperature are shown in Figure 3. The key data are presented in Table 3, which also provides information on the Cr/Mn leaching rate. The physical properties of FS20 and TS85 (pyroxene ceramics) were superior to those of FS80 and TS25 (spinel ceramics) (Table 3); in particular, FS20 and TS85 exhibited higher bending strengths (ISO 13006: 2012: ≥35 MPa) and lower water absorptions. However, FS80 and TS25 had wider sintering temperature ranges and lower Cr/Mn leaching rates than those of FS20 and TS85. The Cr/Mn leaching concentrations of FS20, FS80, TS25 and TS85 met the GB 8978-1996 limits. The densification of ceramic samples sharply decreased their water absorptions (Figure 3), which was beneficial for improving their bending strengths. Extremely high sintering temperatures produced more pores, which reduced the bending strengths of the samples. Cr/Mn leaching concentrations of all the ceramic samples slightly increased with an increase in the sintering temperature. With an increase in the sintering temperature, the Cr/Mn leaching concentrations decreased, except for the case of FS20, owing to a decrease in the concentration of the spinel phase (Figure 2a).

In addition, the type of raw materials influenced the properties of the ceramic samples. The ceramics fabricated from FS had higher sintering temperatures (FS20: 1240 °C and FS80: 1270 °C) than those generated from TS (TS85 and TS25: 1100 °C). This was because FS contained highly refractory phases (forsterite and spinel), whereas TS comprised glass, which promoted sintering and decreased the required sintering temperature. A comparison of the water absorptions of FS20 (0.56%; 30 wt.% dolomite) and FS80 (2.11%; 0 wt.% dolomite) showed that the presence of highly refractory phases in FS (rather than the decomposition of dolomite) was the main reason for the existence of several pores in these samples. Therefore, the ceramic samples prepared from FS had higher water absorptions (FS20: 0.56% and FS80: 2.11%) than those of the samples fabricated from TS (TS25: 0.21% and TS85: 0.12%). Thus, the presence of highly refractory phases in raw materials requires higher sintering temperatures and, consequently, leads to higher water absorptions of the resulting ceramic samples.

To further confirm the conclusion that pyroxene and spinel phases in ceramics contribute to higher bending strengths and lower Cr/Mn leaching rates, respectively, two series of FS and TS ceramics were investigated, and the results are discussed in the next section.

### 3.2. FS and TS Series

#### 3.2.1. Cr/Mn Leaching Rates and Mechanical Properties

**Figure 5:**
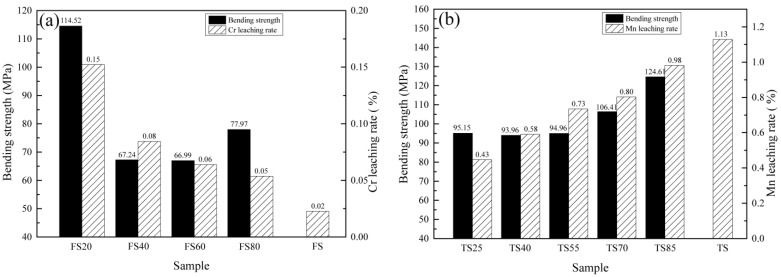
Bending strengths and Cr/Mn leaching rates of the (**a**) FS and (**b**) TS series samples.

The bending strengths and Cr/Mn leaching rates of the FS and TS series samples sintered at optimal temperatures are shown in Figure 5. Among them, the optimum sintering temperature of FS20 was 1240 °C, FS40 was 1250 °C, FS60 and FS80 was 1270 °C and TS25, TS40, TS55, TS70 and TS85 was 1100 °C. FS20 had an optimal bending strength of 114.52 MPa; however, it had the highest Cr leaching rate of 0.15%. From FS20 to FS80, the bending strength initially decreased and then slightly increased, whereas the Cr leaching rate gradually decreased to 0.05% (FS80). Although the leaching concentration of Cr increased with an increase in the amount of FS in raw materials, the leaching concentration of Cr in the cases of the FS series samples met the GB 8978-1996 limit of 1.5 mg/L (Table 4). TS85 had an optimal bending strength of 124.61 MPa; however, it had the highest Mn leaching rate of 0.98%, which was slightly lower than that of TS (1.13%). From TS85 to TS25, the bending strength decreased and remained in the 93.96–95.15 MPa range when the amount of TS in the raw materials was less than 55%. Furthermore, the Mn leaching rate decreased to 0.43% (TS25). The leaching concentrations of Mn for the TS series samples met the GB 8978-1996 limit (2.0 mg/L) (Table 5).

#### 3.2.2. Comparison of Crystals Phases

**Figure 6:**
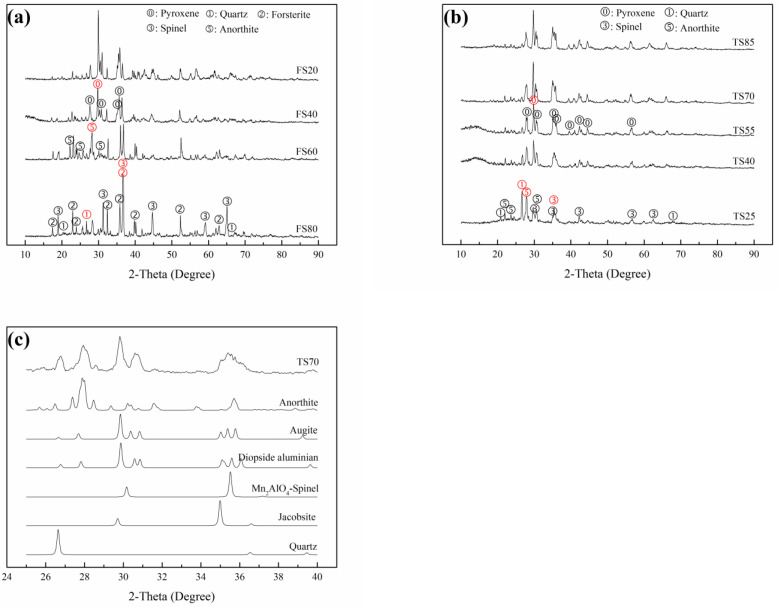
XRD patterns of the (**a**) FS and (**b**) TS series samples sintered at optimal temperatures and XRD pattern (2θ: 25°–40°) of (**c**) TS70 sintered at an optimal temperature.

The XRD patterns of the FS and TS series samples sintered at optimal temperatures are shown in Figure 6. From FS20 to FS80, the amounts of quartz, forsterite, and spinel [Mg (Al, Cr)_2_O_4_] increased, whereas the amounts of pyroxenes, including diopside and augite, decreased (Figure 6a). Anorthite, which was the primary phase in FS60, was a transitional phase between the spinel phase and the pyroxene phase arising from the decrease in the Al_2_O_3_ content. It was deduced that various phases in FS60 and pores originating from the decomposition of dolomite and the inertia of FS led to the lowest bending strength of FS60 (66.99 MPa). Pyroxene was the main phase in FS20, and its concentration substantially decreased from FS20 to FS40, which decreased the bending strength. Moreover, the amounts of spinel phases increased from FS20 to FS80, and there was a corresponding gradual decrease in the Cr leaching rate. 

From TS85 to TS25, overall, the amounts of pyroxene phases decreased, whereas the amounts of spinel phases increased. The pyroxene phases included augite, diopside manganoan and diopside aluminum, which arose from diopside manganoan with an increase in the Al_2_O_3_ content. The spinel phases comprised MnAl_2_O_4_ and jacobsite. MgAl_2_O_4_ was not observed, and some Mg and Si formed pyroxene phases. Spinel and anorthite became the main crystalline phases of TS25 with an increase in the Al_2_O_3_ content. Figure 6c shows the XRD pattern (2θ: 25°–40°) of TS70 and the differences between the diffraction patterns of the pyroxene and spinel phases, and the changes in these patterns can be noticed. Similar to the cases of FS series samples, the amounts of pyroxene phases in the TS series samples were reduced, decreasing the bending strengths of these samples. However, the amounts of spinel phases increased, thereby decreasing the Mn leaching rates. 

The combination of the observed properties and the XRD patterns of the FS and TS series samples further supported the conclusion that pyroxene and spinel phases in ceramics contribute to higher bending strengths and lower Cr/Mn leaching rates, respectively.

#### 3.2.3. Relationship between Crystals Containing Cr/ Mn and Its Retention Ability

**Figure 7:**
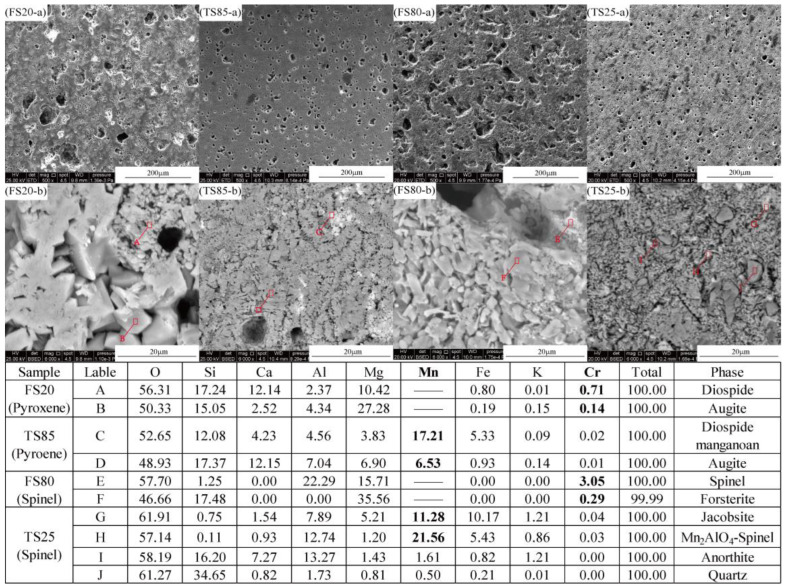
SEM-EDS microstructures of FS20, TS85, FS80 and TS25 sintered at optimal temperatures.

The microstructures of the pores and phases and the presence of Cr/Mn in the ceramic samples were further investigated using SEM-EDS. The SEM-EDS results for FS20, FS80, TS25 and TS85 are shown in Figure 7. Pyroxene (A, B, C, and D) and spinel (E, G, and H) phases were detected in FS20, FS80, TS25 and TS85. These observations are consistent with the XRD results, which provide additional evidence for the conclusion that the pyroxene phase or the spinel phase can be generated as the primary crystalline phase in ceramics by controlling the composition of the raw material and sintering temperature.

FS20 and TS85 were prepared by designing the compositions of raw materials in the crystallization regions of pyroxene phases. FS20 and TS85 had high bending strengths of more than 100 MPa, which were similar to those of the reference samples (107–143 MPa) [7]. Moreover, TS85 had a slightly higher bending strength (124.61 MPa) than that of FS20 (114.52 MPa) because of fewer and smaller pores in it than those in FS20 (Figure 7). These fewer and smaller pores in TS85 were due to the promotion of sintering by the glass phases in TS. In a comparison between the pores in the microstructures (× 500) of FS20 (containing 20% FS and 30 wt.% dolomite) and FS80 (containing 80% FS and 0 wt.% dolomite), we see that FS80 exhibited larger and more pores than those of FS20. This further verified that the main cause of the existence of numerous pores in these ceramic samples was the inertia of FS instead of the decomposition of dolomite. Spinel was the main crystal in FS, and it remained unchanged in ceramics FS80 during the sintering process, as shown in Figure 2b. Different coefficients of thermal expansion, high softening points and in-reactivity with the other raw materials contributed to the formation of pores.

The SEM-EDS results (Figure 7) revealed that certain amounts of Cr and Mn were retained in the pyroxene and spinel phases. The spinel phases contained higher amounts of Cr than those in pyroxene phases, which resulted in the lowest Cr leaching rate of FS80 in the FS series samples. In contrast, higher amounts of Mn were retained in spinel phases than those in pyroxene phases, mainly augite and some amount of diopside manganoan in TS85, and TS25 had the lowest Mn leaching rate. Thus, the spinel phases in ceramics lead to lower Cr/Mn leaching rates because of the retention of higher amounts of Cr/Mn in them than those in pyroxene phases.

Therefore, the optimum designs of the compositions of ceramics from different slags depend on the required mechanical or environmental properties and can be achieved by controlling the formation of main crystals in the ceramic samples. For example, by changing the proportions of the pyroxene and spinel phases, ceramics with both high strengths and stable retentions of heavy metal ions can be obtained.

## 4. Conclusions

FS and TS are two typical slags that contain high contents of Cr_2_O_3_ (3.88 wt.%) and MnO (18.69 wt.%), respectively. In this study, the production of ceramic materials from these metallurgical slags was investigated, and the Cr/Mn leaching behaviors, mechanical properties and phases/microstructures of these materials were evaluated.

The ceramic samples separately prepared from 20–80% FS and 25–85% TS exhibited high bending strengths (77.97–124.61 MPa) and low leaching concentrations of Cr (0.836–1.148 mg/L) or Mn (0.016–0.121 mg/L). FS80 and TS85 had acceptable properties.

Pyroxene/spinel phases were produced as the main crystalline phases in the fired ceramic samples by controlling the compositions of raw materials and the sintering temperature. Pyroxene phases contributed to higher bending strengths (FS20: 114.52 MPa and TS85: 124.61 MPa), whereas spinel phases led to lower Cr/Mn leaching rates (FS80: Cr 0.05% and TS25: Mn 0.43%).

The optimum designs of the compositions of ceramics from different slags depend on the required mechanical or environmental properties and can be achieved by controlling the formation of primary crystals in the ceramic samples. For instance, the proportions of the pyroxene and spinel phases can be changed to obtain ceramics with both high strengths and the stable retention of heavy metal ions. This study provides an important basis for long-term research into the large-scale reuse of heavy metal-containing slags in the ceramic industry.

## Figures and Tables

**Table 1:** Chemical compositions of ferrochrome slag and tundish slag (wt.%).

Raw Materials	CaO	SiO_2_	Al_2_O_3_	MgO	Fe_2_O_3_	MnO	TiO_2_	K_2_O	Na_2_O	Cr_2_O_3_	Total
Ferrochrome slag	3.48	32.32	19.19	38.32	1.48	0.00	0.54	0.15	0.16	3.88	99.53
Tundish slag	16.08	42.79	8.78	7.55	2.32	18.69	1.50	1.39	0.44	0.00	99.54

**Table 2:** Proportions of the raw materials (wt.%).

Sample	Corresponding Slag	Clay	Dolomite	Quartz	Feldspar
FS20	20	40	30	5	5
FS40	40	30	20	5	5
FS60	60	20	10	5	5
FS80	80	10	0	5	5
TS25	25	50	—	—	25
TS40	40	40	—	—	20
TS55	55	30	—	—	15
TS70	70	20	—	—	10
TS85	85	10	—	—	5

**Table 3:** Properties of pyroxene/spinel ceramics.

Properties	Pyroxene Ceramics		Spinel Ceramics	
	FS20	TS85	FS80	TS25
Bending strength (highest)	114.52 MPa	124.61 MPa	77.97 MPa	95.14 MPa
Water absorption (lowest)	0.56%	0.12%	2.11%	0.21%
Sintering temperature	1240 °C	1100–1120 °C	1240–1270 °C	1080–1110 °C
Cr/Mn leaching concentration	0.836 mg/L (Cr)	0.121 mg/L (Mn)	1.148 mg/L (Cr)	0.016 mg/L (Mn)
Cr/Mn leaching rate	0.15% (Cr)	0.98% (Mn)	0.05% (Cr)	0.43% (Mn)

**Table 4:** Cr concentration in leaching solution from FS series samples (mg/L).

Sample	FS	Sample Sintered at Optimal Temperature				Limitations
		FS80	FS60	FS40	FS20	
Cr	0.645	1.148	1.028	0.911	0.836	1.5

**Table 5:** Mn concentration in leaching solution from TS series samples (mg/L).

Sample	TS	Sample Sintered at Optimal Temperature					Limitations
		TS25	TS40	TS55	TS70	TS85	
Mn	0.164	0.016	0.034	0.059	0.082	0.121	2.0

## Data Availability

Data sharing is not applicable to this article.
